# Trajectory and Trend of Weight Status, Emotional Wellbeing and Sleep From Infancy to Childhood to Adolescence in Scotland: An Analysis of Growing up in Scotland Birth Cohort 1

**DOI:** 10.1111/ijpo.70049

**Published:** 2025-08-13

**Authors:** Emma Louise Gale, Joanne Elizabeth Cecil, Andrew James Williams

**Affiliations:** ^1^ School of Medicine University of St Andrews St Andrews Scotland, UK; ^2^ School of Health in Social Sciences University of Edinburgh Edinburgh Scotland, UK

## Abstract

**Background:**

Childhood obesity interventions often overlook sleep and emotional wellbeing, though research shows both are associated with weight status across childhood. The timing of their co‐development and the most effective point for intervention remain poorly understood. The aim of this study was to examine the trajectories of sleep, weight status and emotional wellbeing using the Growing Up in Scotland birth cohort 1 dataset.

**Methods:**

This study conducted secondary data analyses from sweeps 1–10 (10 months‐14 years). Sleep was assessed through main‐carer and self‐reports, covering duration, bedtime, fragmentation, insomnia symptoms and oversleeping. Weight status was evaluated using BMI percentiles from objective height and weight measurements. Emotional wellbeing was evaluated using the emotional symptoms subscale of the Strengths and Difficulties Questionnaire. Trajectories were categorised as stable, improving, or declining for wellbeing; stable, obesogenic or leptogenic for weight; and compared against age‐specific recommendations for sleep.

**Results:**

Analyses from 4157 participants (50.2% male) showed that sleep duration declined with age, falling significantly below age‐specific recommendations between 8 and 14 years. Bedtimes became later and more variable between 8 and 10 years, with insomnia symptoms and delayed sleep onset common by age 14. Obesogenic or fluctuating weight trajectories were observed in 51.2% of participants. Emotional wellbeing declined notably between 10 and 14 years.

**Conclusions:**

Declines in sleep and emotional wellbeing coincided with rising obesity rates between ages 10 and 12. Targeted intervention between ages 8 and 10 years offers a critical opportunity to mitigate risks of obesity, poor sleep and declining emotional wellbeing before adolescence.

## 1 Introduction

Obesity remains a global public health crisis, with worldwide prevalence doubling since 1990 [1]. In 2022, 43% of adults globally were overweight, and 16% were obese, with the associated healthcare costs and economic burden estimated to account for approximately 3% of the global Gross Domestic Product annually [2]. This figure is projected to rise to 3.5% (£3.4 trillion) by 2035, highlighting the growing economic impact of obesity on healthcare systems and productivity [2]. Reducing obesity rates by just 5% could save an estimated £337.5 billion annually by 2060 [3]. In Scotland, the situation is particularly concerning, with 30% of children (up to 16 years old) and 66% of adults classified as overweight or obese, exceeding global averages [4].

Despite ongoing interventions aimed at improving diet and physical activity [5, 6, 7], childhood obesity rates continue to rise [2]. The persistence of rising rates despite public health campaigns indicates a need to expand interventions beyond traditional approaches. Traditional approaches fail to address key social, economic, psychological and environmental determinants, such as sleep and emotional wellbeing [8, 9]. Emerging research emphasises the role of sleep and emotional wellbeing in weight regulation, highlighting the need for integrated strategies [10].

Recent research provides compelling evidence for the role of sleep in weight regulation [11, 12]. Systematic review evidence has demonstrated that pre‐sleep behaviours—such as poor sleep hygiene, inconsistent bedtime routines, a later chronotype, delayed sleep onset and social jetlag—are significantly associated with obesity in adolescents [11]. Poor emotional wellbeing, including depression, anxiety, stress, psychological distress and eating disorders, has also been identified as shared determinants of both poor sleep and obesity in adolescents [13]. Bidirectional relationships between poor sleep, emotional wellbeing and obesity have been documented across diverse populations through multiple mechanisms [14, 15]. For example, disrupted or insufficient sleep can impair metabolic and hormonal processes, including dysregulation of leptin and ghrelin [16, 17], which influence appetite and satiety [18], and increased cortisol levels [19], which contribute to stress and weight gain [20]. Additionally, poor emotional wellbeing may lead to maladaptive coping behaviours such as emotional eating [21, 22], disrupted sleep patterns [23] and reduced physical activity [24], creating a feedback loop that exacerbates both sleep disturbances and obesity [13]. Conversely, obesity and related metabolic dysfunctions can impair sleep quality and duration through mechanisms such as sleep apnoea [25] and inflammation [26] while also negatively impacting mental health due to stigma and body image concerns [27]. Such interconnected pathways highlight the complexity of these relationships and the need for integrated intervention strategies.

To effectively integrate sleep and emotional wellbeing into obesity‐focused interventions, it is essential to examine the trajectories and trends of these factors across childhood and adolescence. Existing longitudinal trajectory research examining sleep, weight status and emotional wellbeing in isolation has shown that sleep duration typically declines [28], emotional wellbeing fluctuates [29], particularly in early adolescence and BMI follows distinct growth curves [30]. However, to our knowledge, no studies have mapped trends in these three domains concurrently within the same population. This study therefore seeks to describe patterns in these health indicators across childhood and adolescence within a single nationally representative cohort.

The aim of this research was to identify the trajectories and trends of sleep, obesity and emotional wellbeing between the ages of 4 and 14 years using data from the Growing Up in Scotland (GUS) birth cohort 1 [31]. Although this study does not directly assess interactions between sleep, emotional wellbeing and obesity, previous research has demonstrated that these outcomes are interrelated, with shared determinants and bidirectional associations across childhood and adolescence [11, 13, 32]. Understanding their co‐developmental patterns can support the design of timely, integrated interventions [13]. Analysing the overlap in the decline of health along these trajectories will help identify critical periods for intervention. This research can guide the development of targeted, multi‐faceted interventions designed to address the complex interplay between sleep, emotional wellbeing and obesity, ultimately helping to combat all three components simultaneously.

## 2 Materials and Methods

### 2.1 Ethics Statement

This study received ethical approval from the School of Medicine Ethics Committee at the University of St Andrews, operating under the authority of the University Teaching and Research Ethics Committee (UTREC; Approval Code: MD16516, granted on 29 September 2022). The data used were obtained under a Special Licence from the UK Data Service (dataset SN 5760, Project 226 100) and were fully anonymised and de‐identified before access.

### 2.2 Study Design, Recruitment and Data Collection

The GUS study is a long‐term research project tracking children's health, behaviour and growth from birth to early adolescence, beginning at 10 months old (2005/06) and continuing until they are 14 years old (2018/19). Children and their primary caregivers were randomly selected from Child Benefit records by HM Revenue and Customs and invited to join the study [33]. The initial cohort included 5271 children and their caregivers in Scotland [33]. In 2018, 502 more participants were added to increase the sample size (boost sample) [34]. By phase nine, the retention rate was 56%, which decreased to 51% by phase ten, including the additional participants [34]. The initial data collection and sweeps up to 8 were approved by the Scotland ‘A’ Medical Research Ethics Committee (MREC) (application reference 04/M RE 1 0/59), while sweeps 9 and 10 were approved by the NatCen Research Ethics Committee [35].

The inclusion criteria for the GUS study were: (i) children born between June 2004 and May 2005, (ii) families living in a Scottish local authority. For the boost sample, additional inclusion criteria were: (i) mothers aged 16–24 at the child's birth, and/or (ii) residing in the 15% most deprived areas according to the 2016 Scottish Index of Multiple Deprivation (SIMD) [34]. Only one child per household was selected, giving twins an equal chance of inclusion [34].

During each sweep, measures were recorded, including physical measurements and interviews with the main carer, partner, teacher or child, as well as use of linked digital health records. Not all interviews and measures were repeated in every sweep, and new measures were added in later sweeps [36, 37, 38, 39, 40, 41, 42, 43, 44, 45]. From sweeps 1 to 9, trained interviewers conducted in‐home interviews using laptops, consisting mostly of quantitative closed questions [33, 46, 47, 48, 49, 50, 51, 52, 53]. In sweep 10, due to the COVID‐19 pandemic, data collection shifted: from initially face‐to‐face (January to March 2020) to telephone or web (March to December 2020) [34]. The main carer, usually the birth mother, completed a questionnaire in all ten sweeps [35]. Interviews averaged 60–80 min, covering socio‐environmental and demographic details about the carer, household and child [36, 37, 38, 39, 40, 41, 42, 43, 44, 45]. Early sweeps focused on birth measures and early milestones, while later sweeps addressed the child's behaviour, wellbeing, diet, physical activity, social development and school and home life [36, 37, 38, 39, 40, 41, 42, 43, 44, 45].

## 3 Measures

### 3.1 Demographics

#### 3.1.1 Child‐Specific Demographics

In all sweeps (1–10), the main carer reported the child's age and sex [36, 37, 38, 39, 40, 41, 42, 43, 44, 45]. In sweep 10, children reported their puberty status through gender‐specific questions [45]. Male‐specific questions included: ‘Have you noticed a deepening in your voice?’ and ‘Has hair begun to grow on your face?’ if the child answered yes to either of those questions, they were then asked at what age they noticed the change [45]. Female‐specific questions included ‘have you ever menstruated (had your period)?’ if the child answered yes, they were then asked at what age the first menstruation occurred [45].

#### 3.1.2 Caregiver‐Specific Demographics

From sweeps 2 to 10, the main carer reported the ethnicity and highest education level of both themselves and their partner [36, 37, 38, 39, 40, 41, 43, 44, 45] Ethnicity and highest level of education at baseline were used in these analyses. Socioeconomic status (SES) was evaluated across sweeps 1 to 10 using the National Statistics Socio‐Economic Classification (NS‐SEC), which considers the work status and profession of the main carer or partner [54]. The NS‐SEC categories range from ‘managerial and professional’ to ‘never worked’, with each assigned a corresponding number [36, 37, 38, 39, 40, 41, 42, 43, 44, 45]. Households with complete NS‐SEC data over seven sweeps had their socioeconomic status trajectories analysed. Cases with a standard deviation (SD) below 0.4 were labelled ‘static’, while those above indicated overall ‘increasing’ or ‘decreasing’ trends.

### 3.2 Anthropometrics

The child's body mass index percentile (BMIp) was calculated using weight (kg) divided by height (m^2^), measured by trained professionals in sweeps 4 and 6–10 [36, 37, 38, 39, 40, 43, 45]. Some, but not all, weight status measures in sweep 10 were self‐reported due to the COVID‐19 pandemic restricting researcher‐participant in‐person engagement. Weight status was categorised into five groups using International Obesity Task Force cut‐offs based on Centers for Disease Control percentiles [55]: underweight (< 5th percentile), healthy weight (5th ≤ to < 85th percentile), overweight (85th ≤ to < 95th percentile), obese (95th ≤ to < 98th percentile) and morbidly obese (≥ 98th percentile) [34, 36, 37, 38, 39, 40, 43, 45, 46, 47, 48, 49, 50, 52].

Child BMIp trajectory was determined by comparing changes across six sweeps (sweeps 4 and 6–10). A ±10 percentile change per year in BMI was used to categorise weight trajectories. This threshold was selected to differentiate between expected growth variation and more substantial changes in weight status over time, allowing for the identification of obesogenic and leptogenic patterns. A change less than ±10 percentiles per year was labelled ‘normal growth’. A decrease of more than 10% per year was termed ‘leptogenic’, while an increase of more than 10% per year was termed ‘obesogenic’. Missing data were filled by comparing the adjacent time points. If data were missing at the end or for three consecutive time points, the BMIp trajectory was marked as missing. In total, there were eight distinct categories within the BMIp trajectory (Table 1).

**TABLE 1:** BMI percentile status trajectory category definitions.

BMIp status trajectory	Definition
Normal growth	All 4 or 5 changes were no marked change, that is they stayed on a BMIp
Slight leptogenic	One of the 4 or 5 changes was leptogenic, all others no marked change
Early leptogenic	Two or more of the first three changes in sequence were leptogenic
Late leptogenic	Two or more of the last three changes in sequence were leptogenic
Leptogenic	No overall pattern but mostly leptogenic
Fluctuating	Similar numbers of leptogenic and obesogenic changes
Slight obesogenic	One of the 4 or 5 changes was obesogenic, all others no marked change
Early obesogenic	Two or more of the first three changes in sequence were obesogenic
Late obesogenic	Two or more of the last three changes in sequence were obesogenic
Obesogenic	No overall pattern but mostly obesogenic

Abbreviation: BMIp, body mass index percentile.

### 3.3 Sleep

Sleep patterns were assessed through questions to the main carer regarding the child participant's bedtime consistency, sleep fragmentation and duration [36, 37, 38, 41, 42, 44, 45]. In later sweeps, the child participant also answered questions about their own sleep, including fragmentation, how long it took to fall asleep, presence of insomnia, excessive sleep and overall sleep duration (Table 2) [36, 37, 38, 41, 42, 44, 45]. Sleep duration trends were assessed by comparing reported sleep duration at each age against age‐specific sleep recommendations, classifying participants as meeting, exceeding, or falling below recommended sleep durations at each time point.

**TABLE 2:** Assessment of sleep outcomes.

Topic	Question	Responses	Reported	Sweep(s)
Bedtime	“What time does ^ChildName go to sleep?”	Open response (24 h)	Main carer	5, 6, 7, 8
Bedtime regularity	“On weekdays during term time, does ^ChildName go to bed at a regular time?”	“Always” (1) “Usually” (2) “Sometimes” (3) “Never” (4)	Main carer	5, 6, 7
Sleep fragmentation	Experienced “waking up too much or too early”	“Yes” (1) “No” (0)	Child participant	10
Sleep onset latency	“How long does it take you to fall asleep”	“0–15 min” (1) “16–30 min” (2) “31–45 min” (3) “46–60 min” (4) “More than 60 min” (5)	Child participant	10
Insomnia symptoms	Experienced “insomnia” (no definition provided)	“Yes” (1) “No” (0)	Child participant	10
Oversleeping	Experienced “sleeping too much”	“Yes” (1) “No” (0)	Child participant	10
Sleep duration	“Roughly how many hours sleep would ^ChildName tend to get in a typical 24 h period, including any sleeps or naps during the day”	Open response (hours)	Main carer	1, 3, 6, 7
	“How many hours sleep do you get on a school night?”	Open response (hours)	Child participant	10
	“How many hours sleep do you get on a non‐school night?”	Open response (hours)	Child participant	10

### 3.4 Emotional Wellbeing

Emotional wellbeing of the child participant was assessed using the emotional symptoms subscale of the Strengths and Difficulties Questionnaire (SDQ) [56], across six timepoints: ages 4 (Sweep 4), 6 (Sweep 6), 8 (Sweep 7), 10 (Sweep 8), 12 (Sweep 9) and 14 years (Sweep 10). The data were parent reported in sweeps 4 and 6–9 [36, 37, 38, 40, 43, 44] and child‐reported in sweep 10 [45]. The higher the score, the poorer the child's emotional wellbeing.

The Growing Up in Scotland study does not follow a strictly annual schedule, and timepoints were selected based on the availability of comparable SDQ data. For this paper, change was assessed across five developmental intervals: 4–6, 6–8, 8–10, 10–12 and 12–14 years. To determine wellbeing trajectories, changes in SDQ scores across six sweeps (sweeps 4, 6–9 and 10) were assessed. A ±10% change in SDQ emotional symptom scores between sweeps was used to classify trajectory direction. This threshold was chosen to capture changes (increasing, decreasing or static) over time. The participants were classified into three categories at each time point: (1) ‘decrease’ if their SDQ score improved by more than 10% from the previous time point, (2) ‘static’ if their SDQ score remained within ±10% and (3) ‘increase’ if their SDQ score worsened by more than 10%. For inclusion in the trajectory analyses, participants needed at least three data points between ages 4 and 14 years. Missing data were handled by carrying forward values from the nearest adjacent time points where possible. If data were missing at the final time point or for three consecutive sweeps, the trajectory was marked as missing.

### 3.5 Data Analyses

Data were imported and analysed using SPSS Version 28. Descriptive statistics were used to summarise participant characteristics. For variables that were normally distributed, results are reported as mean ± standard deviation. Little's MCAR test was conducted to assess the randomness of missing data [57]; listwise deletion was applied for incomplete cases.

Trajectory categories for weight status, and emotional wellbeing and the trend for sleep duration were assigned based on pre‐defined rules outlined in the respective methods sections. Frequencies and proportions were calculated to describe the distribution of participants across trajectory categories. Patterns were examined to identify periods of notable change. As the primary aim of this study was to examine descriptive patterns and trajectories over time, no covariate adjustments were made. This approach allowed a focus on the overall developmental trends in health outcomes, rather than the contribution of individual demographic or socioeconomic factors.

## 4 Results

### 4.1 Missing Data

The MCAR test [57] result (*x* ^2^ = 1746.67, dF = 6802, *p* = 0.01) suggests that the likelihood is that the missing values were not missing at random. Missing data were addressed using listwise deletion.

### 4.2 Sample Characteristics

The overall sample included 4157 participants. The child participant sample was 50.2% male (Table 3). Caregiver ethnicity was 96.4% white and 3.6% other background (Table 3). Mean BMI (kg/m^2^) of the caregiver was 26.87 ± 5.51 kg/m^2^ and the most common education level of the caregiver was up to 16–18 years (66.5%), followed by under 16 years (15.4%) and 19–24 years (15.0%) (Table 3). The most frequently reported NS‐SEC group was ‘managerial and professional occupation’ (57.6%), followed by ‘intermediate occupations’ (14.3%) and ‘semi‐routine or routine occupations’ (12.5%).

**TABLE 3:** Sample characteristics.

		Sample characteristics (*N* = 4157)			
		*N*	%	Mean	SD
Child gender	Male	2085	50.2		
	Female	2072	49.8		
Caregiver ethnicity	White	3044	96.4		
	Other ethnic background	113	3.6		
Caregiver BMI		3081		26.9	5.5
Caregiver education level	Under 16 years	84	15.4		
	16–18 years	363	66.5		
	19–24 years	82	15.0		
	25 years or older	14	2.6		
	Still in full‐time education	3	0.5		
Caregiver NS‐SEC	Managerial and professional occupations	1818	57.6		
	Intermediate occupations	453	14.3		
	Small employers/self‐employed	221	7.0		
	Lower supervisory and technical occupations	240	7.6		
	Semi‐routine and routine occupations	394	12.5		
	Never worked	31	1.0		
Caregiver NS‐SEC trajectory	Static	2788	88.3		
	Decrease	189	6.0		
	Increase	180	5.7		

Abbreviations: BMI – body mass index; *N* – number; NS‐SEC – National Statistics Socio‐economic Classification; SD – standard deviation.

### 4.3 Sleep Trend Across Childhood and Adolescence

#### 4.3.1 Sleep

##### 4.3.1.1 Sleep Duration

Mean sleep duration was 12.9 ± 1.7 h at 10 months, 11.5 ± 1.4 h at 3 years, 10.8 ± 1.0 h at 6 years, 10.4 ± 1.0 h at 8 years and 7.9 ± 1.1 h at 14 years (Table 4).

**TABLE 4:** Summary characteristics of sleep.

Sleep outcome		Sample characteristics			
		*N*	%	Mean	SD
Bedtime regularity (5 years)	Always	2639	68.9		
	Usually	946	24.7		
	Sometimes	131	3.4		
	Never	115	3		
Bedtime regularity (6 years)	Always	2318	63.4		
	Usually	1109	30.3		
	Sometimes	154	4.2		
	Never	76	2.1		
Bedtime regularity (8 years)	Always	1951	56.5		
	Usually	1281	37.1		
	Sometimes	144	4.2		
	Never	78	2.3		
Bedtime (5 years)		3716		19:50	00:40
Bedtime (6 years)		3581		19:57	00:35
Bedtime (8 years)		3375		20:19	00:33
Bedtime (10 years)		3146		21:05	01:04
Delayed sleep onset (14 years)	0–15 min	1023	37		
	16–30 min	993	35.9		
	31–45 min	385	13.9		
	46–60 min	203	7.3		
	60 min+	162	5.9		
Insomnia symptoms (14 years)	Yes	539	75.2		
	No	178	24.8		
Through the night (3 years)	Never	487	11.6		
	1–2 times a week	412	9.8		
	3–5 times a week	680	16.2		
	6 times a week	648	15.5		
	Every night	1963	46.8		
Through the night (6 years)	Never	137	3.7		
	1–2 times a week	157	4.3		
	3–5 times a week	307	8.4		
	6 times a week	424	11.6		
	Every night	2632	72		
Through the night (8 years)	Never	91	2.6		
	1–2 times a week	83	2.4		
	3–5 times a week	149	4.3		
	6 times a week	267	7.7		
	Every night	2864	82.9		
Sleep duration (hours) (10 months)		5208		12.9	1.7
Sleep duration (hours) (3 years)		4197		11.5	1.4
Sleep duration (hours) (6 years)		3656		10.8	1
Sleep duration (hours) (8 years)		2674		10.4	1
Sleep duration (hours) (14 years)		2351		7.9	1.1
Sleep fragmentation (14 years)	Yes	171	23.8		
	No	546	76.2		
Oversleeping (14 years)	Yes	226	31.5		
	No	491	68.5		

Abbreviations: Mins – minutes; *N* – number; S – sweep; SD – standard deviation; SN – school night; WE – weekend.

##### 4.3.1.2 Sleep Problems in Childhood

A regular bedtime was reported for 93.6% of 5‐year‐olds, 93.7% of 6‐year‐olds and 93.6% of 8‐year‐olds (Table 4). Average bedtime and its variability increased with age: 19:50 ± 0:40 at 5 years, 19:57 ± 0:35 at 6 years, 20:19 ± 0:33 at 8 years and 21:04 ± 1:04 at 10 years (Table 4). The percentage of participants sleeping through the night (≥ 6 nights per week) rose with age, from 59.3% at 10 months to 62.3% at 3 years, 83.6% at 6 years and 90.7% at 8 years (Table 4).

##### 4.3.1.3 Sleep Problems in Adolescence

At 14 years, 75.2% of participants reported insomnia, 23.9% reported waking up too much or too early, and 27.1% experienced a sleep onset latency of 30 min or more (Table 4).

#### 4.3.2 Sleep Trend

**FIGURE 1:**
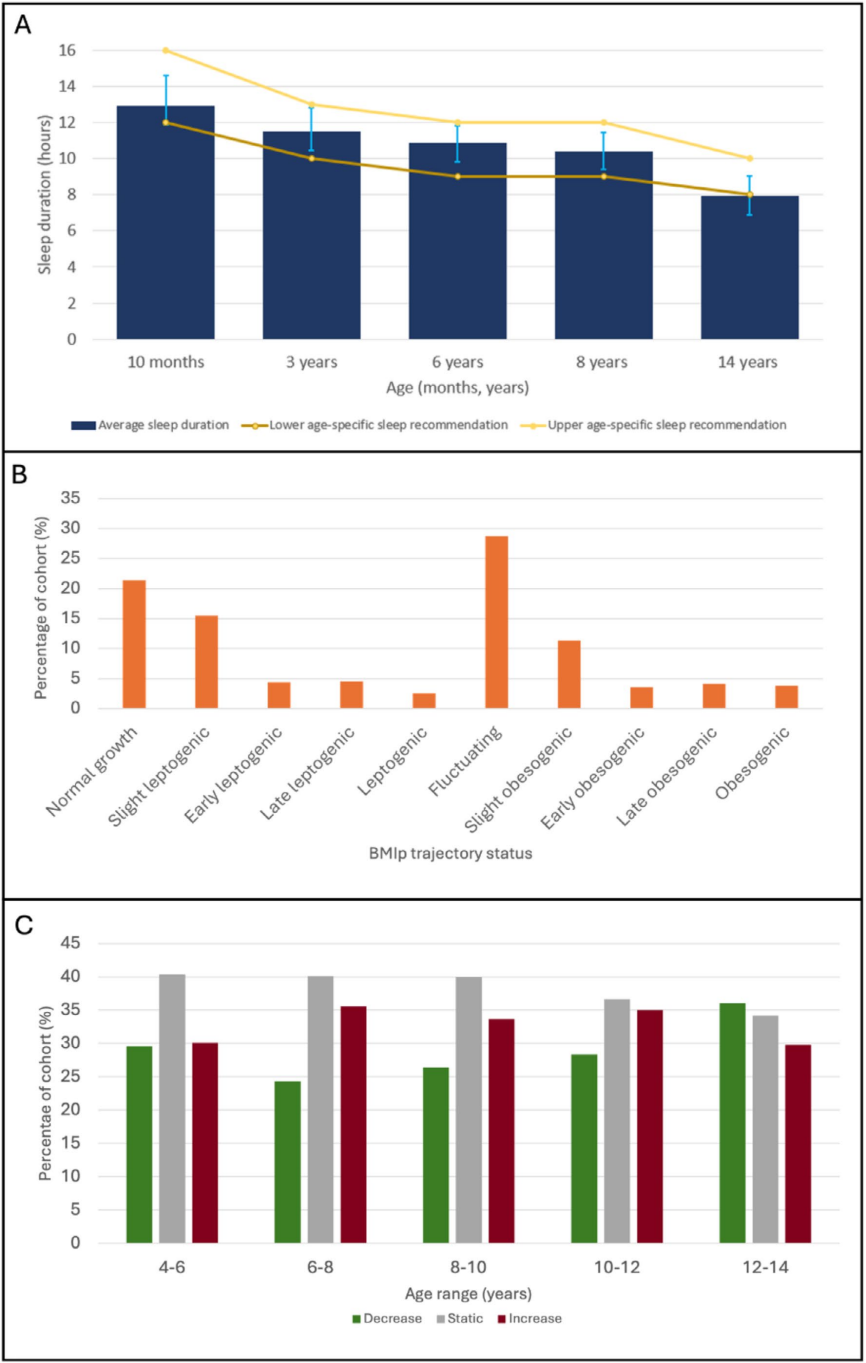
Trajectories of sleep, weight status and emotional wellbeing. (A) Trajectories of sleep duration and the meeting of sleep recommendations between 10 months and 14 years old; (B) Weight status trajectories between 4 and 14 years; (C) Trajectories of emotional wellbeing between 4 and 14 years old.

With increasing age, the ability to sleep through the night improves. Mean sleep onset time and the variability (SD) in reported sleep onset times also increase. Wake time remained relatively stable across age groups. Mean sleep duration stayed within the recommended guidelines [58] until 8 years but fell below the recommendations by 14 years (Figure 1a).

### 4.4 Weight Status Trajectory Across Childhood and Adolescence

#### 4.4.1 Weight Status

Mean child BMIp (±SD) was recorded at 4‐years (0.7 ± 0.3), 6‐years (0.7 ± 0.3), 8‐years (0.7 ± 0.3), 10‐years (0.7 ± 0.3), 12‐years (0.7 ± 0.3) and 14‐years (0.6 ± 0.3) (Table 5).

**TABLE 5:** Body mass index percentile reported between 4 and 14 years old.

BMIp	Sample characteristics			
	*N*	%	Mean	SD
4 years old	3665		0.7	0.3
6 years old	3483		0.7	0.3
8 years old	2636		0.7	0.3
10 years old	2554		0.7	0.3
12 years old	2597		0.7	0.3
14 years old	2056		0.6	0.3

Abbreviations: BMIp – body mass index percentile; *N* – number; SD – standard deviation.

#### 4.4.2 Weight Status Trajectory

Fluctuating or obesogenic growth patterns account for 51.5% of the sample. Trajectory categories are shown in Figure 1b.

### 4.5 Emotional Wellbeing Trajectory Across Childhood and Adolescence

#### 4.5.1 Emotional Wellbeing

Mean child emotional symptoms moderately consistent between 4 and14 years old: 4‐years (1.2 ± 1.4), 6‐ years (1.2 ± 1.6), 8‐years (1.4 ± 1.7), 10‐years (1.6 ± 1.8), 12‐years (1.8 ± 2.1) and 14‐years (1.8 ± 2.1) (Table 6). However, with age, variability in the reported emotional wellbeing score increased.

**TABLE 6:** Emotional symptoms between 4 and 14 years old.

Emotional symptoms (years)	Sample descriptives			
	*N*	%	Mean	SD
4 years old	3974		1.2	1.4
6 years old	3634		1.2	1.6
8 years old	3427		1.4	1.7
10 years old	2589		1.6	1.8
12 years old	2598		1.8	2.1
14 years old	2342		1.8	2.1

Abbreviations: *N* – number; SD – standard deviation.

#### 4.5.2 Emotional Wellbeing Trajectory

Emotional wellbeing trajectories were classified based on individual changes in SDQ scores at each time point (Figure 1c). A stable or improving wellbeing trajectory was most observed between ages 6 and 10 years. Emotional wellbeing began to decline from age 10, and by ages 12 to 14, a worsening trajectory became more prevalent than stable or improving wellbeing.

## 5 Discussion

### 5.1 Overall Findings

This study identified overlapping patterns in the trajectories of sleep duration, weight status and emotional wellbeing from childhood to adolescence. Sleep duration decreased with age, falling below recommended levels between 8 and 14 years, alongside later and more variable bedtimes. Over half of the sample exhibited obesogenic or fluctuating BMI trajectories, and emotional wellbeing declined notably between 10 and 14 years. While this study did not test for statistical associations between the trajectories, these concurrent trends suggest that the period after age 8, particularly between 10 and 12 years, may be a key window for intervention. This age range represents a time when multiple aspects of child health are shifting, indicating the potential value of targeting sleep, weight and wellbeing in a more integrated and preventative manner.

### 5.2 Sleep Trend Across Childhood and Adolescence

The findings from these analyses indicated that child bedtime, bedtime regularity and sleep duration remained consistent and within sleep recommendations up to 8 years of age. After 8 years, there was a shift towards shorter sleep duration (by 14 years), later bedtimes (by 10 years) and increased bedtime variability (by 10 years). Additionally, the proportion of adolescents experiencing sleep fragmentation, insomnia symptoms and prolonged sleep onset latency at 14 years was higher than the proportion of children who struggled to sleep through the night or had poor bedtime regularity at 8 years. These changes may be partially attributed to hormonal shifts during puberty, which cause a delay in circadian rhythms towards a later chronotype. This shift, combined with adherence to an early chronotype schedule (e.g., early school start times), contributes to reduced sleep duration [59]. Supporting recent evidence comes from a cross‐sectional study of Chinese adolescents, which found that younger age at menarche was significantly associated with shorter sleep duration and later bedtimes [60]. A causal relationship between later age of menarche and longer sleep duration from the longitudinal ‘Children of 1997’ birth cohort study has also been reported [61]. These data suggest that insufficient or later sleep during pre‐adolescence may influence precocious puberty.

From analyses of the GUS dataset, short sleep duration as early as 10 months and later bedtimes from 5 to 8 years were associated with shorter sleep duration and later bedtimes at 14 years. This pattern suggests that poor sleep may persist as a chronic issue across childhood and adolescence. Previous studies support this finding [62, 63, 64]. For example, a longitudinal study assessing the relationship between sleep problems in childhood and short sleep duration during the transition from adolescence to adulthood reported that more frequent and severe sleep problems at 7–9 years of age were associated with shorter sleep duration at 16–19 years [63]. While GUS data do not extend beyond 14 years, this evidence suggests that chronic sleep problems established during childhood may persist into adulthood.

The initial pubertal changes occurring between 8 and 10 years may act as an important period when shifts in bedtime and reduced sleep duration begin to manifest. This age range could therefore represent an effective window for interventions aimed at mitigating the negative impacts of pubertal changes on sleep and related outcomes, such as obesity.

### 5.3 Weight Status Trajectory Across Childhood and Adolescence

The current findings from the GUS analyses revealed that 22.8% of children had an obesogenic BMIp trajectory (including slightly obesogenic, early obesogenic, late obesogenic and obesogenic) and 29.7% of children had a fluctuating BMIp trajectory. As BMIp trajectories are primarily derived from longitudinal studies, they are less commonly reported in the literature, making direct comparisons challenging. However, using individual BMIp time point measures, findings from ‘Obesity Action Scotland’ (2021) reported that 24.1% of Primary 1 pupils (aged 4–5 years) were at risk of being overweight or obese, with obesity rates in children and adolescents continuing to rise annually [65]. Additionally, ‘The Scottish Health Survey’ in 2021 reported an increase in the percentage of boys at risk of obesity from 16% (2–6 years) to 27% (7‐11 years) and an increase in the percentage of girls at risk of being overweight from 8% (2–6 years) to 12% (7–11 years), indicating an increase in those at risk of obesity across childhood and adolescence [66]. Data from other longitudinal studies have provided insight into overweight trajectories in adolescence. For example, analyses of data from the American multisite Study of Early Child Care and Youth Development revealed chronic obesity was prevalent in 28.4% of 9 to 15 years [67]. However, obesity is known to be more prevalent among children and adolescents with vulnerable traits, such as ethnic minorities and lower SES [68, 69, 70, 71]. Children with a lower SES often start early childhood with a higher BMI than those with higher SES [70]. Ethnic minorities, such as Hispanics in the USA, have also been reported as having a higher BMIp and are less likely to have a decreasing or static BMIp than white participants [71].

The current findings suggest that, from ages 4 to 14 years, 21.4% of participants had a stable BMIp trajectory, indicating that their weight status remained consistent relative to the reference growth curves. In contrast, 28.7% exhibited a fluctuating BMIp trajectory, meaning their BMIp deviated from expected growth patterns at multiple time points. Among those with stable BMIp trajectories, the proportion classified as overweight or obese remained consistent over time.

It is important to note that while BMI itself naturally changes with growth, BMIp is a standardised measure designed to account for expected growth patterns relative to a reference population. A stable BMIp suggests that a child is following the same growth pattern as children at the same BMI percentile in the reference population; whereas changes in BMIp indicate deviation from these growth curves. Previous research supports the current findings, suggesting that BMIp can fluctuate during the transition from childhood to adolescence, with deviations from expected trajectories occurring at key developmental stages [67, 68, 69, 70, 72, 73].

Prior evidence has highlighted that shifts in BMIp trajectories occur at key developmental stages, with evidence suggesting an increase around ages 4–5 years and again at approximately 11 years, coinciding with the onset of puberty [69]. These shifts align with what is known about adiposity rebound, a normal phase in child growth that is already accounted for in BMIp reference standards [74, 75, 76, 77]. However, deviations from expected BMIp trajectories may signal periods of increased risk for excessive weight gain. Consequently, pre‐adolescence, particularly just before these key transitions, may represent an optimal window for health‐promoting interventions aimed at stabilising weight status and preventing obesity.

Prior research has indicated that many adolescents, when transitioning to adulthood, have a BMIp that remains constant or increases with age; rarely (2%–8%) does adolescent BMIp decrease going into adulthood when the adolescent is overweight or obese [68]. Thus, it is important to encourage healthy lifestyles and a healthy BMIp in pre‐and early adolescence. In addition to pinpointing the time at which changes in obesity occur across childhood and adolescence, it is also important, as with poor sleep trajectories, to identify potential modifiable behavioural causes of the fluctuation seen in growth.

### 5.4 Wellbeing Trajectory Across Childhood and Adolescence

The analyses of the GUS data identified a decline in emotional wellbeing between 10 and 14 years, consistent with research highlighting adolescence as a critical period for mental health challenges. Emotional wellbeing has been shown to worsen with age, peaking in early adolescence, driven by puberty, academic pressures and social changes [78]. Recent cohorts show sharper declines compared with earlier generations, potentially reflecting societal shifts such as increased screen time, reduced physical activity and perceived greater stress levels [79, 80]. These findings underscore the need to address wellbeing during this vulnerable developmental stage.

The findings of this study suggest that wellbeing trajectories remain stable during early childhood, with declines emerging after age 8. This contrasts with some existing research highlighting earlier emotional difficulties [79, 80], potentially due to differences in measurement methods or the buffering effects of parental support during childhood. However, some studies have indicated a start in decline around the age of 11 and a peak of poor mental health by 14–15 years [81, 82]. In our analyses, a modest increase in emotional wellbeing scores was observed at age 14 years. However, this may partly reflect a shift in informant, as parent‐report measures were used up to age 12, with self‐report measures introduced at age 14 years. This change in reporting method should be considered when interpreting the apparent improvement at this time point.

The simultaneous decline in wellbeing, shorter sleep duration and increasing obesity suggests key opportunities for targeted interventions. For children between ages 8 and 10 years, efforts should focus on addressing early signs of emerging issues, including stress management, sleep hygiene and coping skills, to prevent deterioration. Interventions during ages 10 to 12 should prioritise managing existing difficulties and mitigating reinforcing behaviours. By recognising emotional wellbeing as both a driver and outcome of poor sleep and obesity, interventions can adopt a holistic approach to improving adolescent health.

### 5.5 Optimal Age for Health‐Promoting Interventions

Based on the findings of this study and existing research, sleep problems are most prevalent between the ages of 8 and 14 years, obesity rates peak between 4 and 5 years and 10 and 12 years and poor emotional wellbeing is most common between 10 and 14 years. The overlap of these health challenges occurs primarily between 10 and 12 years. Therefore, proactive health‐promoting interventions should be targeted at children aged 8 to 10 years to mitigate the development of poor sleep, obesity and poor emotional wellbeing between 10 and 12 years of age.

## 6 Strengths and Limitations

The scope of the present paper was to descriptively map health trends across childhood and adolescence. A major strength of this work is the use of the GUS dataset, the only longitudinal birth cohort dataset in Scotland for which analyses of sleep and obesity trajectories over time in childhood are possible. The dataset includes a large sample of over 5000 participants in the original cohort, providing robust data for longitudinal analyses. Another key strength is the breadth of health‐related questions, which allowed for comprehensive analyses across multiple domains, including obesity and emotional wellbeing. These outcomes were recorded consistently across sweeps, enabling detailed trajectory analyses from childhood to adolescence.

However, there are some limitations that should be acknowledged. Variability in participant attendance across sweeps, particularly during the COVID‐19 pandemic, resulted in a reduced number of valid records available for analysis. Many individuals who participated in earlier and mid‐sweeps did not continue to Sweep 10, leading to non‐random missing data. As a result, the final analytical sample may not fully reflect the original GUS cohort or the broader Scottish adolescent population. Although the GUS study was designed to be nationally representative, cumulative attrition over time may have introduced selection bias. This should be acknowledged when interpreting the findings, as differences between retained participants and those lost to follow‐up could impact the observed relationships. Additionally, while obesity and emotional wellbeing were consistently recorded, sleep outcomes were not measured consistently, with a significant gap in sleep data between ages 8 and 14 years. This gap limited the precision of identifying when sleep deterioration occurs within this critical period. Finally, sleep and emotional wellbeing were based on self‐ or parent‐reports, which may be influenced by recall bias or subjective interpretation. Some subjective sleep measures, notably the presence of insomnia and sleep fragmentation, included single questions with a binary response without providing definitions for insomnia and fragmentation (Table 2), which may have introduced variability in interpretation and potentially inflated prevalence estimates. The absence of objective measures (e.g., actigraphy or clinical assessments) may limit the precision of the reported trajectories.

## 7 Conclusion

Sleep duration and emotional wellbeing showed clear declines between the ages of 8 and 14 years, while weight status became increasingly variable, with a rise in obesogenic and fluctuating trajectories. The ages of 8 to 10 years represent a potential target for health‐promotion interventions that address early signs of emerging issues or potential behaviours and determinants contributing to the development of poor sleep, obesity and emotional wellbeing. Interventions during 10 to 12 years should focus on managing emerging or existing issues within these three health domains. These findings highlight the need for targeted, age‐specific strategies to mitigate risks and improve long‐term health outcomes.

## Conflicts of Interest

The authors declare no conflicts of interest.

## Data Availability

The data that support the findings of this study are available from UK Data Service. Restrictions apply to the availability of these data, which were used under license for this study. Data are available from https://ukdataservice.ac.uk with the permission of UK Data Service.
